# Metastatic Squamous Cell Carcinoma Component from an Adenosquamous Carcinoma of the Lung with Identical Epidermal Growth Factor Receptor Mutations

**DOI:** 10.1155/2015/283875

**Published:** 2015-08-23

**Authors:** Jarred Burkart, Konstantin Shilo, Weiqiang Zhao, Efe Ozkan, Amna Ajam, Gregory A. Otterson

**Affiliations:** ^1^Department of Internal Medicine, The Ohio State University College of Medicine, Columbus, OH 43210, USA; ^2^Department of Pathology, The Ohio State University College of Medicine, Columbus, OH 43210, USA; ^3^Department of Radiology, The Ohio State University College of Medicine, Columbus, OH 43210, USA

## Abstract

The case reported is a young “light” ex-smoker who initially had a localized adenosquamous carcinoma bearing an epidermal growth factor receptor (EGFR) sensitizing mutation. He first recurred six months after initial treatment within the brain with a pure squamous histology and the same EGFR mutation. Surgical resection and radiation rendered him disease-free. Subsequent isolated recurrence within the lung eighteen months later was a pure adenocarcinoma, again with the same identified EGFR mutation. These histologic changes (from adenosquamous to pure squamous to pure adenocarcinoma) have been described but not before in the absence of any selection pressure with EGFR tyrosine kinase inhibitors. This case points out the histologic “flexibility” of EGFR mutant lung cancers and the importance for appropriate molecular testing in nonsmokers with lung cancer of any histologic type.

## 1. Introduction

The identification of mutations within the epidermal growth factor receptor (EGFR), and the finding that these mutations make tumors exquisitely sensitive to EGFR tyrosine kinase inhibitors (TKIs), has revolutionized treatment of non-small-cell lung cancer (NSCLC). EGFR mutations are more common in never-smokers, in patients with Asian ethnicity, and in patients with adenocarcinoma histology [1]. We present the case of a young light ex-smoker (1-pack-year history of smoking) with an adenosquamous lung carcinoma that relapsed within the brain. The relapsed lesion showed pure squamous morphology and retained the exon 19 EGFR mutation in the absence of any preceding TKI treatment.

## 2. Case Presentation

**Figure 1:**
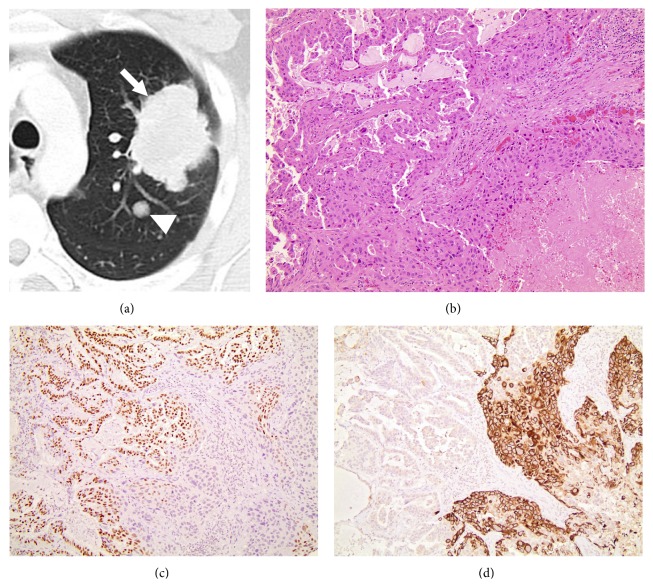
Radiologic and histologic features of lung cancer at initial presentation. (a) Contrast enhanced axial CT image shows a 6 cm lobulated left upper lobe mass (arrow) with irregular margins and pleural tags and a 1 cm satellite left upper lobe nodule (arrowhead). Histological examination shows (b) adenosquamous carcinoma with two distinct tumor components including (c) adenocarcinoma, immunoreactive with thyroid transcription factor-1 and (d) squamous cell carcinoma, immunoreactive with keratin CK5/6.

The patient was a 43-year-old male who felt well until the onset of intermittent hemoptysis. A chest X-ray demonstrated a left upper lobe (LUL) mass. A Computed Tomography (CT) scan of the chest demonstrated a large LUL mass with satellite lesions and a 1 cm left lower lobe (LLL) nodule (Figure 1(a)). No mediastinal adenopathy or extrathoracic disease was noted, confirmed by Positron Emission Tomography (PET). Brain Magnetic Resonance Imaging (MRI) was negative. Following negative mediastinoscopy, the patient underwent a LLL segmental resection and LUL lobectomy, showing an adenosquamous carcinoma (Figures 1(b)–1(d)) with EGFRExon 19 in-frame (18 bp) deletion. This was detected using a Polymerase Chain Reaction- (PCR-) based Fluorescence Fragment Analysis assay. He received four cycles of adjuvant cisplatin and docetaxel for pT4N0 disease.

**Figure 2:**
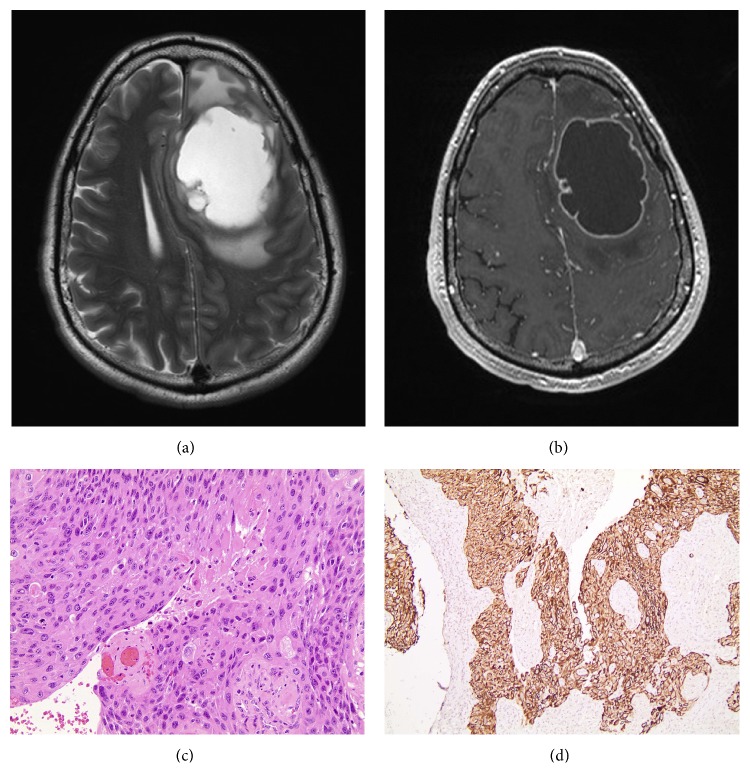
Radiologic and histologic features of brain metastasis. (a) Axial T2 weighted sequence demonstrates a large cystic mass in the left frontal lobe with surrounding edema, mass effect, and midline shift. (b) Axial postcontrasted T1 weighted sequence demonstrates an irregular cystic ring enhancing lesion with surrounding edema and mass effect. (c) Light microscopic examination shows squamous cell carcinoma (d) that is diffusely immunoreactive with keratin CK5/6.

Six months after completion of adjuvant chemotherapy, the patient experienced headaches and altered mental status. A brain MRI showed an irregular frontal lobe lesion measuring 7 by 5 cm (Figures 2(a) and 2(b)). Craniotomy with resection revealed a metastatic poorly differentiated squamous cell carcinoma (Figures 2(c) and 2(d)) harboring the same EGFR mutation as the original tumor. The patient received postoperative focal radiotherapy. Given the absence of systemic recurrence, no additional chemotherapy or EGFR directed therapy was administered.

**Figure 3:**
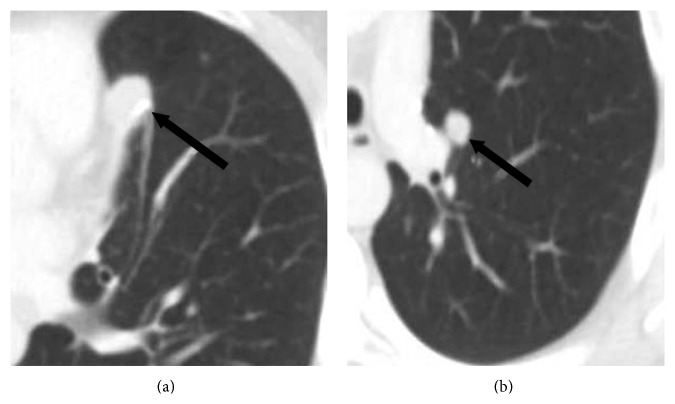
Radiologic features of recurrent malignant lung disease. Contrast enhanced axial CT image shows (a) a 1.9 × 1.4 cm nodular paramediastinal opacity and (b) a left 1.1 × 0.9 cm perihilar nodule. Histological examination demonstrated pure adenocarcinoma with the same EGFR exon 19 mutation that was present in the original tumor.

Approximately eighteen months following resection of the brain metastatic disease, surveillance imaging demonstrated new left lung and hilar nodules (Figures 3(a) and 3(b)). The patient underwent left pneumonectomy. Histology demonstrated invasive mucinous adenocarcinoma (without squamous component) with the same EGFR exon 19 mutation. At this time, mutation analysis was completed using a next generation sequencing assay performed on the Ion AmpliSeq Cancer Hotspot Panel. The patient is currently in surveillance.

## 3. Discussion

Adenosquamous carcinomas are uncommon primary lung tumors. A report in 25 Korean patients with adenosquamous tumors showed that if an EGFR mutation is present (in 44% of their patients), it is present in both components. This finding supports a monoclonal derivation of this tumor (as opposed to a hypothetical “collision” tumor) [2]. An intriguing aspect of our case is the “evolution” of the tumor first to a pure squamous histology in the brain metastatic site in the absence of any selective pressure by an EGFR tyrosine kinase inhibitor followed by a later local recurrence with pure adenocarcinoma histology. Histological transformation in patients with EGFR mutations treated with TKIs is a relatively newly described mechanism of TKI resistance; however, our patient had no prior exposure to TKIs [3].

This report also helps inform the strategy for mutational analysis. While the incidence of “actionable” EGFR or other mutations is infrequent in heavy smokers or squamous histology, the exclusion of these patients from testing would exclude a large proportion of patients with actionable mutations [4]. The National Comprehensive Cancer Network (NCCN) NSCLC and College of American Pathologists (ACP)/International Association for the Study of Lung Cancer (IASLC)/Association for Molecular Pathology (AMP) panels recommend testing in all nonsquamous histologies, in those with a never-smoking or light ex-smoking history, and in those with limited biopsy or cytology specimens. Neither panel excludes the possibility of molecular testing in patients with squamous histology, specifically to address the issues associated with limited biopsy samples and sometimes equivocal nature of histologic diagnosis. Our case highlights these recommendations in the idea that our patient had a light smoking history and if the biopsy had “missed” the adenocarcinoma portion and returned a pure squamous cytology or histology, one might have concluded that the patient was unlikely to have an EGFR mutation.

## 4. Conclusion

This case points out that all nonsmoking lung cancer patients should have molecular testing for “actionable” mutations, regardless of histologic type.
